# Mr. Wright on Medical Reform

**Published:** 1813-12

**Authors:** Robert Wright

**Affiliations:** Memb. Roy. Coll. Surg. London. Whitechapel Road


*To the Editor of the Medical and Physical Journal.*

SIR,

AFTER the energetic language of a Parkinson, and the laudable exertions of a committee, I conceive that those who do not coincide with its views are called upon to give their opinion of the matter in exoneration of themselves, as the withholding the aid called for, and maintaining a total silence, makes a man look very contemptible in the scale of society; and it is this idea that furnishes me with an apology for obtruding myself on the public.

So long as the legislature remains indifferent to the present disgraceful state of medical practice, the exertions of individuals, however laudable, will, I fear, be of little avail; because, unless the state be thoroughly alive to her interests, and active in watching and guarding her own safety, she will never enact laws so seemingly remotely affecting her as to put subjects in a better train for seeking the advice they need. If the learned men of this nation have no idea of promoting the public weal beyond that of their having given support or assent to any discovery that individuals may have promulgated, and awarding according to its merit, they go no further in their line of duty than some of our religionists, who distress the poor on a week-day for a paltry sum, that they may have the better means of giving something at a charity sermon, or enjoy the vanity of seeing their names and donations on the records of a newspaper.

Whenever popular obloquy is aimed at any class of the community by the vulgar, its frequent repetition often causes the higher classes of society to use it with such seriousness, as affixes to it that stamp of veracity which, when traced to its genuine source, too often proves it to be of unshaken stability. Adduce only e. g. the very frequent opprobrium that is levelled at that heterogeneous class of men called "doctors"—"Ah! you give two bottles of stuff to make your patients well, and one to make them *bad* again." Few expressions can surpass the illiberality conveyed by this, though many such are broached by the vulgar, and imbibed and credited by their superiors, till, from a jocular expression, a popular opinion is created, and this, frequent usage renders proverbial, which it never attains unless there be some truth in the remark. Thus then we see that the shafts of wit and the mockery of fools, directed against a divine, a god-like art, are the sad tokens by which is conveyed the deceit and hypocrisy they imagine its professors to be capable of, instead of the veneration and respect in which they ought to be held.

Why

Why is all this? Because no branch of the profession is restrained from infringing on the rights of the other. Hence we see why it is the shopkeeper sells salts, senna, manna, magnesia, rhubarb, and other articles; we see the disregard paid as to whether he sell common salts or saltpetre, till the fatality attending his ignorance is woefully brought home to him: we see the perfumer and the patent medicine vendor (shame to the nation that encourages it!) have their share of a business they must be totally ignorant of; and whether they sell the most actively deleterious substances, under the specious titles quack medicines assume, or a walking cane or pomade divine, they are regardless of every thing but the profit awaiting them; just like the blind organ player, who rattles out a tune careless about its dissonance, so long as it procures him a few pence. This is not wrong of the persons who keep these shops—self-interest is implanted in us by Nature: still, if the evil be not attacked at its very root (and this I consider to be so), and if medical men will not in one body convene, and address the state to alter the present system, and to prescribe the limits each branch should enjoy, wrangling, cross-purposes, and petty meannesses will still pervade the body it infests, and no solid advantages will be derived while each is alike eager for aggrandizement. As it now stands, the shopkeeper, the perfumer, and the patent medicine vendor usurp the province the chemist and druggist ought in a great measure to exercise; the druggists step into the department of the surgeon-apothecary, who, unable to remedy this from any appeal to his College, whose duty it is to protect him, oversteps the sphere of his action into that of the physician,—who, being alike unable from any appeal to his College to obtain redress, by reason of its not being able or willing, or being shamefully negligent, he is compelled to fight his own battles in his own way, and as is his disposition so is the nature of his quarrels, either sly, aggravating, or malignant. Others are doubtless equally in fault, but I only speak of the latter because that is the ultimatum from whence repulsion (if I may be allowed to call it so) takes place.

Thus then (and sad is the observation in a country like this) we see that those who should most strive to cement an union are most at variance, whilst the neutrality of the quack shelters him; and the opprobrium which should be cast at him flies off obliquely to the medical practitioner, who, belonging to a body, exerts no influence, or they might pronounce "Cuique sua dos est," by allotting to nine-tenths of them a halter, and to the rest, by strenuously endeavoring to procure for them from government a pension proportionate to their discovery, and its influence on the public weal. That man whose patient investigation and deep research discovers any thing whereby the miseries "that flesh is heir to" can be eradicated, is as much a hero as he whose consummate skill destroys fleets, besieges towns, or drives the enemy back to his own territory; but the man that will not promulgate this for the benefit of the public at large, when a suitable remuneration is offered for the discovery, deserves as much the detestation of his species as a murderer would call for from a hero.

It is quackery, hydra-headed quackery, that must most strenuously be attacked. To the honor of the nobility of this nation, I cannot but believe that were a subscription opened for the accumulation of vast sums to compensate for the disclosure of the most noted of these compositions to a select body of competent judges, we should see miseries relieved that before were incurable, contention would be quelled, and happiness restored. When I see or hear of any eminent man candidly giving sanction to a useful preparation, I can draw no other conclusion than the above, and can have no wish more ardent for my fellow creatures than that of seeing it accomplished. We are told that Daffy's Elixir is Tr. Sennæ comp. in. Sp. Vin. Gallici, with Spanish liquorice; Soloman's Balm of Gilead—Tr. Cantharides, with Bals. Copaivæ; Scott's Pills—Alöes and Ol. Anisi; Eau Medicinale—white helebore wine:—then surely it must be a desideratum that medical men should have, and only have, these substances to use according to the circumstances of the case.

But I will not occupy too much space in your useful miscellany. I wish to induce some gentleman more competent than myself, to discuss the following question, viz. Can any solid benefit be obtained whilst quackery is daily increasing?—a fact, I believe, pretty generally allowed, and, of which, being so well convinced, I cannot but say that were a society established for the extermination of this murdering practice, I would willingly give as many pounds as I should reluctantly give farthings to the other, where, in their proposed amendment, the consideration of quackery is totally omitted.

Receive, sir, or reject these remarks, according to your idea of their utility. I should like to sign "Medio;" but if you wish my name, I have no reason to conceal it, remaining, sir, your constant reader,

ROBERT WRIGHT,

*Memb. Roy. Coll. Surg. London.*

*Whitechapel Road,*

*Oct.* 13, 1813.

